# An update regarding the role of WNK kinases in cancer

**DOI:** 10.1038/s41419-022-05249-y

**Published:** 2022-09-19

**Authors:** Mengxi Xiu, Li Li, Yandong Li, Yong Gao

**Affiliations:** Department of Oncology, Shanghai East Hospital, Tongji University School of Medicine, 200120 Shanghai, China

**Keywords:** Cancer, Targeted therapies, Oncogenes, Tumour-suppressor proteins

## Abstract

Mammalian WNK kinases (WNKs) are serine/threonine kinases that contain four members, WNK1–4. They function to maintain ion homeostasis and regulate blood pressure in mammals. Recent studies have revealed that the dysregulation of WNKs contributes to tumor growth, metastasis, and angiogenesis through complex mechanisms, especially through phosphorylating kinase substrates SPS1-related proline/alanine-rich kinase (SPAK) and oxidative stress-responsive kinase 1 (OSR1). Here, we review and discuss the relationships between WNKs and several key factors/biological processes in cancer, including ion channels, cation chloride cotransporters, sodium bicarbonate cotransporters, signaling pathways, angiogenesis, autophagy, and non-coding RNAs. In addition, the potential drugs for targeting WNK-SPAK/OSR1 signaling have also been discussed. This review summarizes and discusses knowledge of the roles of WNKs in cancer, which provides a comprehensive reference for future studies.

## Facts

- The expression of WNK kinases is dysregulated in several types of cancer.
- WNK kinases are involved in the regulation of tumor growth, metastasis and angiogenesis through complex mechanisms.
- WNK-SPAK/OSR1 signaling is a potential target for cancer treatment.

## Open questions

- What are the functional differences or similarities between different WNK kinases in cancer?
- What is the relationship between different phosphorylation sites and the functions of WNK kinases in cancer?
- How to target WNK-SPAK/OSR1 signaling in cancer more effectively and safely?

## Introduction

**Fig. 1:**
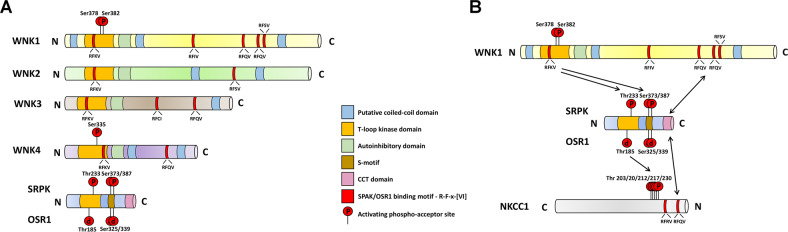
Functional analysis of structural domains recognized in WNK kinases and their substrates. **A** Structures of WNK kinases and SPAK/OSR1. WNKs contain a homologous T-loop kinase domain, an autoinhibitory domain, two coiled-coil domains and R-F-x-[VI] motifs. Known essential phosphorylated-serine residues for WNK activation are Ser-335 (for WNK4) and Ser378/382 (for WNK1). SPAK and OSR1 contain a homologous T-loop kinase domain, a S-motif and a CCT (conserved carboxyl-terminus) domain. Phosphorylated-threonine residues in their kinase domain (Thr-185 in OSR1 and Thr-233 in SPAK) are essential for their activation, while phosphorylated-serine residues in their s-motif (Ser-325/339 in OSR1 and Ser-373/387 in SPAK) are non-activation sites. **B** Domains and sites essential for the WNK-SPAK/OSR1-NKCC1 axis (take WNK1 for example). The CCT domain of SPAK/OSR1 binds to R-F-x-[VI] motifs in WNK1 and NKCC1. WNK1 activates SPAK/OSR1 by phosphorylating on Thr-185 (for OSR1) or Thr-233 (for SPAK). Activated SPAK/OSR1 then phosphorylates and activates NKCC1.

With-no-lysine [K] kinases (WNKs) are a family of serine/threonine protein kinases, and they are featured by the unusual placement of a catalytic lysine residue (Lys233) in their ATP binding site [1]. In mammals, there are four WNKs (WNK1, WNK2, WNK3 and WNK4) that share 85% homology over their kinase domains (Fig. 1A) [1, 2]. The gain-of-function mutation of *WNK1* and *WNK4* causes an autosomal-dominant disease pseudohypoaldosteronism type II, which is characterized by hypertension and hyperkalemia [3]. Among the best-described targets of WNKs are kinase substrates SPS1-related proline/alanine-rich kinase (SPAK) and oxidative stress-responsive kinase 1 (OSR1) [2, 4, 5]. SPAK and OSR1 are two mammalian protein kinases in the STE protein kinase subfamily. WNKs phosphorylate these kinases and activate them, thereby regulating the activities of cation-chloride-cotransporters (CCCs) that maintain ion homeostasis throughout the body, such as NKCC1 [2, 4, 5]. Domains and sites essential for the WNK-SPAK/OSR1-NKCC1 axis are shown in Fig. 1B.

Like other protein kinases, WNKs are closely linked to the pathological processes of several diseases and they are potential drug targets [6–8]. However, the functions and druggability of WNKs remain unclear due to the fact that not all kinase family members are included in kinome screening panels and there has been an underinvestment in these targets [9–11]. Therefore, a comprehensive understanding of the biological roles and druggability of WNKs in a specific disease is essential.

In recent years, the aberrant expression of WNKs is found in human cancers, as shown in several studies [12–17]. In addition, WNKs may serve as clinical biomarkers, whose expression in cancer tissues is related to the prognosis of cancer patients. For example, high expression of WNK1 can predict poor overall survival (OS) in patients with hepatocellular carcinoma (HCC) and colorectal cancer (CRC), and is correlated with clinicopathological parameters such as high pathological grade and advanced clinical stage [14, 17]. In contrast, low expression of WNK2 is significantly associated with early tumor recurrence and poor OS in HCC patients [18].

In this review, we mainly focus on describing the confirmed and/or unconfirmed mechanisms by which WNKs are involved in cancer progression, including the relationships between WNKs and ion channels, CCCs, sodium-bicarbonate cotransporters (NBCs), signaling pathways, angiogenesis, autophagy and non-coding RNAs (ncRNAs) in cancer. In addition, we also discuss known drugs that block WNK-OSR1/SPAK signaling in cancer.

## WNKs modulate ion channels, CCCs, and NBCs in cancer

### Ion channels

**Fig. 2:**
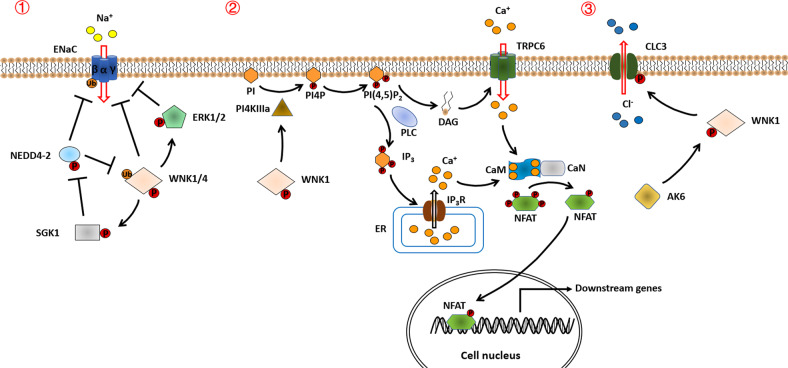
Potential molecular mechanisms by which WNKs regulate ion channels in cancer cells. ① WNK1/4 can activate ENaC-induced sodium influx by phosphorylating and activating SGK1, and WNK4 can inhibit ENaC-induced sodium influx by decreasing ENaC expression on the cell membrane or activating ERK1/2 signaling. NEDD4-2 can ubiquitinate ENaC and WNK4 to inhibit their activity. ② WNK1 induces TRPC6-mediated cation influx by recruiting PI4KIIIa to the plasma membrane. Mechanistically, PI4KIIIa induces PI4P synthesis and further promotes PLC-mediated PI(4,5)P_2_ hydrolysis and production of IP_3_/DAG. IP_3_ activates IP_3_R on the endoplasmic reticulum, resulting in stored Ca^2+^ release into the cytoplasm. DAG activates the TRPC6 channel to promote cation influx. Increased intracellular Ca^2+^ then activates CaN by binding to CaM. Activated CaN dephosphorylates NFAT and promotes its nuclear translocation. NFAT promotes the transcriptional activity of downstream tumor-related genes and contributes to cancer progression. ③ AK6 facilitates the phosphorylation of WNK1 to activate CLC3 upon hypotonic conditions. Increased chloride efflux leads to regulatory volume decrease and cancer cell shrinkage and invasion/migration.

Channel-mediated ion transports participate in the regulation of the cell behaviors of normal and cancer cells, such as cell motility, survival, death, migration, and invasion [19, 20]. Indeed, ion channels localizing in the plasma membrane can sense and respond to changes in the extracellular environment, and they are involved in several signaling pathways during cancer progression [19, 20]. WNKs are important kinases that can phosphorylate several ion channels and may regulate their activity in cancer, including the amiloride-sensitive epithelial sodium channel (ENaC), transient receptor potential canonical channel 6 (TRPC6) and chloride channel 3 (ClC3) (Fig. 2).

### ENaC

ENaC (encoded by the *SCNN1* gene family) is a heterotrimeric ion channel that is made up of three subunits (αβγ) [21]. ENaC functions to promote sodium reabsorption across epithelial tissues, which is crucial for extracellular volume homeostasis and the control of blood volume/pressure [22, 23]. In addition, ENaC can regulate cell shape by mediating cell mechanosensing and influencing actin cytoskeletal architecture, thus affecting cell behaviors such as cell migration [24–26].

The high expression of α-ENaC or γ-ENaC in human gliomas is wildly found to promote cancer progression [27–29]. Mechanistically, the sodium gradient established by ENaC-induced sodium influx enables glioma cells to recover cell volume and further promotes cell migration and invasion, and these effects can be reversed through knockdown of α-ENaC or γ-ENaC [28]. In breast cancer (BC), the upregulation of γ-ENaC can induce a chronic inflammatory response that promotes cancer progression [30]. In contrast, another ENaC subunit α-ENaC was found to have an anti-cancer effect that decreases the proliferation of BC cells [31].

WNKs have a complex relationship with ENaC (Fig. 2). WNK1 and WNK4 can promote ENaC activity via activating serum and glucocorticoid-induced protein kinase 1 (SGK1), a protein kinase that is able to control ion channels and CCCs [32, 33]. And this involves inhibitory phosphorylation of the ubiquitin protein ligase neural precursor cell expressed developmentally downregulated protein (NEDD)4-2 [32, 33]. However, SGK1 and NEDD4-2 are also found to inhibit ENaC activity by phosphorylating (SGK1) or ubiquitinating (NEDD4-2) WNK4 [34, 35]. In addition, WNK4 can also negatively regulate ENaC by enhancing ENaC internalization and retrograde trafficking or activating the ERK1/2 MAPK signaling [36, 37]. Considering that several findings have revealed the key roles of ENaC, SGK1, NEDD4-2, and ERK during cancer progression [27–31, 38–40], WNKs may also regulate ENaC in cancer, which needs further explosion.

### TRPC6

TRPC6 (encoded by the gene *TRPC6*) is a receptor-activated nonselective cation channel that plays an oncogenic role by inducing cation influx and intracellular Ca^2+^ signaling in cancer cells [41–44]. In kidney cancer, WNK1 is found to activate TRPC6-induced Ca^2+^-NFAT signaling, which promotes the proliferation and migration of cancer cells [45]. Mechanistically, WNK1 can induce the synthesis of PIP_2_ through stimulating PI4KIIIa activity and potentiating G_q_-PLC signaling to activate the TRPC6-Ca^2+^-NFAT axis [45, 46]. The details of the mechanism by which WNK1 regulates TPRC6 are shown in Fig. 2.

### CLC3

CLC3 belongs to the voltage-gated CLC family and it functions to induce chloride efflux and initiate a regulatory volume decrease (RVD) in response to cell swelling upon hypotonic conditions [47, 48]. Through this mechanism, CLC3 is involved in cancer progression by controlling cancer cell shrinkage during invasion [49]. In human testicular cancer cells, the activity of volume-regulated chloride channel CLC3 is regulated by WNK1, which is activated by adenylate kinase 6 (AK6) [50] (Fig. 2). Mechanistically, AK6 can directly bind to WNK1 protein and induce the phosphorylation of WNK1 on residue Thr-60. Knockdown of *AK6* can decrease the expression of WNK1 and CLC3, resulting in increased intracellular Cl^−^ concentration in cancer cells upon hypotonic conditions [50]. The dysregulation of CLC3-controlled RVD can inhibit cancer cell proliferation and induce cell apoptosis, indicating the critical role of the AK6-WNK1-CLC3 axis during cancer progression [50].

### CCCs

**Fig. 3:**
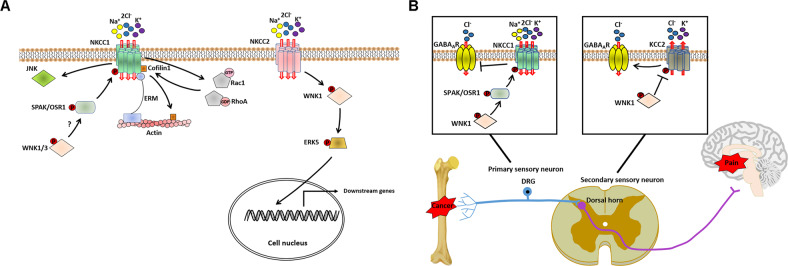
Potential molecular mechanisms of the relationship between WNKs and CCCs in cancer. **A** WNK1/3 is activated in cancer cells in response to stimuli such as temozolomide-induced intracellular Cl^−^ loss and apoptotic volume decrease, followed by activation of NKCC1 through (or without) phosphorylation and activation of SPAK/OSR1. On the one hand, NKCC1 increases Na^+^/K^+^/Cl^−^ influx and induces regulatory volume increase to maintain cancer cell survival. And on the other hand, NKCC1 promotes cancer cell migration by activating MAPK-JNK signaling and driving cytoskeleton organization through interacting with actin-regulating proteins ERM and Cofilin-1 and activating RhoA/Rac1 signaling pathways. NKCC2 can instead activate WNK1/ERK5 signaling to upregulate the expression of cell proliferation-related genes. **B** WNK1 contributes to bone cancer pain by activating SPAK/OSR1–NKCC1 axis in primary sensory/dorsal root ganglion (DRG) neurons while inhibiting KCC2 expression in second sensory/dorsal horn post-synaptic neurons. NKCC1 activation and KCC2 inhibition reduce Cl^−^ extrusion and lead to pathological γ-aminobutyric acid (GABA)-induced depolarization in neurons.

Studies have shown that WNKs can modulate CCCs in cells through SPAK and OSR1 [4, 51, 52]. Normally, WNKs in mammalian cells are phosphorylated and activated in response to volume perturbations or osmotic stress, and they subsequently bind to and phosphorylate SPAK/OSR1 [52]. Activated SPAK/OSR1 in turn phosphorylate and activate Na^+^-driven, Cl^−^ influx CCCs (Na^+^-K^+^-2Cl^−^-cotransporter isoform (NKCC1-2)) and inhibit K^+^-driven, Cl^−^ efflux CCCs (the K^+^-Cl^−^ cotransporter isoform (KCC1-4)) in cells, which shrunken cells return to normal volume [4]. So far, relationships between WNKs and NKCC1-2/KCC2 in cancer have been revealed (Fig. 3).

### NKCC

NKCCl (encoded by the gene *SLC12A2*) is a key CCC protein that is activated by WNK-SPAK/OSR1 signaling and maintains cell volume homeostasis. NKCCl is wildly overexpressed in several cancers, such as gliomas, gastric cancer (GC) and esophageal squamous cell cancer [12, 53–56]. In gliomas, temozolomide (TMZ)-mediated chemotherapy can trigger a significant loss of intracellular K^+^/Cl^−^ and apoptotic volume decrease (AVD), leading to cell death of cancer cells [12, 53]. However, TMZ treatment can also induce the activation of WNK1-OSR1 signaling in glioma cells, which activates NKCC1 activity and then in turn accumulates intracellular Na^+^/K^+^/Cl^−^ to counteract AVD [12, 53]. In addition to WNK1, WNK3 can also maintain NKCC1-mediated transport thereby inducing regulatory volume increase (RVI) in glioma cells [54]. However, it is unclear whether WNK3 regulates NKCC1 through a direct physical interaction or through activating SPAK and/or OSR1 [54].

WNK-NKCC1 signaling can promote cancer cell migration [12, 53, 54, 57]. One mechanism is that WNK1-OSR1 signaling induces interactions between NKCC1 and ezrin-radixin-moesin (ERM) proteins [12]. ERM proteins are responsible for the linkage of the actin cytoskeleton to membrane proteins, and the activation of ERM proteins may stimulate cytoskeletal rearrangements and further induce glioma cell migration [12]. Recent studies also reveal that NKCC1 regulates the actin cytoskeleton and promotes the invasiveness of gliomas through increasing the expression of Cofilin-1 protein and its activators Rho GTPases RhoA and Rac1 [58, 59]. In GC, the overexpression of NKCC1 contributes to the proliferation, migration and invasion of cancer cells via activating MAPK-JNK signaling [55]. Knockdown of *NKCC1* in cancer cells can inhibit the expression of EMT-related proteins (MMP2/9, Snail and vimentin) and increase the expression of anti-EMT protein E-cadherin [55, 60]. However, other downstream molecules/pathways of the WNK-SPAK/OSR1-NKCC1 axis in tumors have not been elucidated.

In contrast to NKCC1, NKCC2 (encoded by the gene *SLC12A1*) acts as an upstream activator of WNK1 and it is overexpressed in HCC due to histone methylation within its promoter region [61]. NKCC2-activated WNK1 can phosphorylate ERK5 and activate ERK5 MAPK signaling, further supporting liver tumorigenesis by upregulating the expression of cell proliferation-related genes *c-Fos*, *c-Jun*, *c-Myc*, and *Cyclin D1*. Treatment of SLC12A1 antagonist Bumetanide can inhibit HCC growth both in vitro and in vivo, indicating the targeted therapeutic value for blocking the NKCC2–WNK1–ERK5 axis [61].

### KCC2

KCC2 (encoded by the gene *SLC12A5*) is an electroneutral and neuronal-specific member of CCCs. Within neurons, NKCC1 and KCC2 serve as two main regulators of synaptic inhibition or hyperpolarization through regulating the inhibitory neurotransmitters γ-aminobutyric acid (GABA) receptors [62]. NKCC1 upregulation and/or KCC2 downregulation can result in the accumulation of intracellular Cl^-^ in dorsal horn (DH) post-synaptic neurons, which inhibits GABA-mediated hyperpolarization/inhibition and leads to hyperalgesia and allodynia following nerve injury or peripheral inflammation [63].

In neurons, KCC2 can be phosphorylated by WNK-SPAK/OSR1 signaling, which has an inhibitory effect on KCC2 activity and Cl^−^ extrusion [64, 65]. Gao et al. have found that WNK1-induced NKCC1/KCC2 dysregulation contributes to bone cancer pain (BCP), one of the most common symptoms of cancer-induced pain and affects one-third of cancer patients [66]. In a rat model of BCP, WNK1 expression is overexpressed in the DH and dorsal root ganglion (DRG) neurons, which upregulates NKCC1 in the DRG and decreases KCC2 expression in the DH [66]. Knockdown of *WNK1* by intrathecal siRNA can inhibit the protein expression of SPAK/OSR1 in the DRG, but not in the DH [66]. These findings are consistent with the results of a previous study that knockdown of the *Wnk1/Hsn2* isoform in a mouse model of neuropathic pain significantly reduced the phosphorylation of KCC2 without affecting the expression levels of phosphorylated-NKCC1 and SPAK/OSR1 in the spinal cord [67].

Although WNK1 may not induce the inhibitory phosphorylation of KCC2 in the DH through SPAK/OSR1, intrathecal injection of SPAK/OSR1 inhibitor closantel is also found to markedly reduce mechanical hyperalgesia and movement-evoked pain in BCP rats through decreasing NKCC1 expression in the DRG and restoring KCC2 expression in the DH [66]. This may be explained that the activation of the WNK1-SPAK/OSR1-NKCC1 axis in the primary sensory neurons (DRG neurons) is a necessary condition for WNK1-mediated KCC2 inhibition in the second sensory neurons (DH post-synaptic neurons) during the generation of BCP [66].

In a study of 110 osteosarcoma patients, the clinical efficacy of controlled-release morphine tablets in combination with celecoxib and its effect on WNK1 expression have been evaluated [68]. The results exhibit that the application of controlled-release morphine tablets and celecoxib can reduce the pain degree, incidence of adverse events and WNK1 expression in the peripheral blood of patients [68]. This suggests that WNK1 may be a potential indicator of evaluating BCP.

### NBCs

**Fig. 4:**
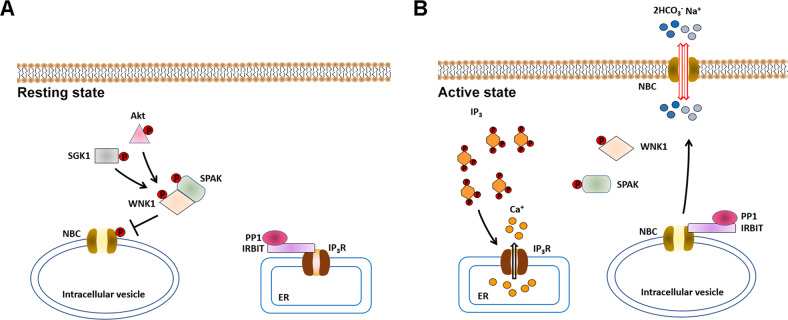
Potential molecular mechanisms by which WNKs regulate NBCs in cancer stem cells (CSCs). **A** In the resting state of CSCs, SGK1/Akt-phosphorylated WNK1 recruits SPAK to induce the inhibitory phosphorylation of NBCs. The IRBIT-PP1 complex binds to IP_3_R and inhibits Ca^2+^ efflux from the endoplasmic reticulum. **B** In the active state of CSCs, the IRBIT-PP1 complex is released from IP_3_R by high levels of IP_3_ and binds to NBCs, antagonizing the effect of WNK1 and increasing the activity of NBCs on the cell surface.

WNKs act as scaffolding proteins rather than kinases to modulate the activity of NBCs [69]. Mechanistically, the N-terminal of WNK1 containing residue Thr-60 recruits SPAK to NBCs in intracellular vesicles, leading to the inhibitory phosphorylation of NBCs and reducing their cell surface expression [70]. However, this effect can be reversed by the activation of IRBIT protein that can recruit PP1 to dephosphorylate NBCs and increase both the cell surface expression and activity of NBCs in cancer stem cells (CSCs) (Fig. 4) [69, 70]. When CSCs are at the quiescent state or intracellular IP_3_ is at low levels, IRBIT interacts with IP_3_ receptors and inhibits their activity. Upon stimulation, increased intracellular IP_3_ in CSCs displaces IRBIT, and IRBIT instead relieves the inhibitory effect of WNK1 on NBCe1 and NBCn1 [70]. Notably, the scaffolding function of WNK1 is dependent on the phosphorylation of residue Thr-60 by Akt and SGK1. Blocking the Akt/SGK1-WNK1/SPAK-NBC axis by DDPM (a cytotoxic compound that can inhibit WNK1 Thr-60/Ser-382 phosphorylation) or Akt/SGK1 inhibitors induces the excessive activation of NBCs especially in quiescent CSC, resulting in dysregulated ion homeostasis and finally cell death [70].

### WNKs and other signaling pathways in cancer

**Fig. 5:**
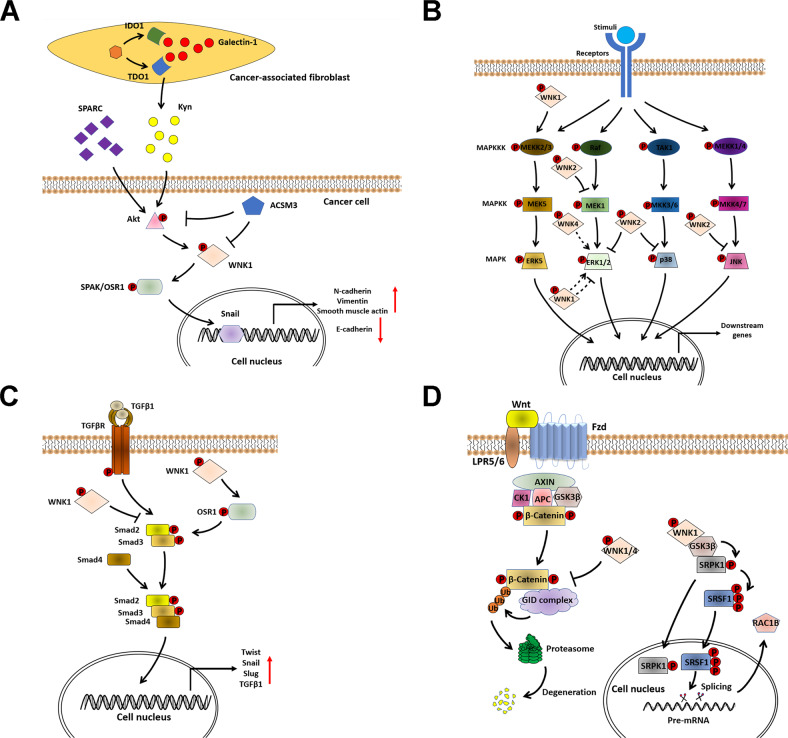
WNKs are associated with Akt, MAPK, TGF-β, GSK3β and Wnt signaling pathways in cancer cells. **A** Galectin-1 promotes the production of Kyn from cancer-associated fibroblasts. SPARC and Kyn in the tumor microenvironment activate Akt-WNK1-SPAK/OSR1 signaling in cancer cells and further promote cancer EMT. ACSM1 can instead inhibit Akt-WNK1 signaling and prevent tumor metastasis. **B** WNK1 activates ERK5 MAPK signaling, while WNK2 inhibits ERK1/2, p38 and JNK MAPK signaling pathways. In addition, WNK1 and WNK4 may potentially regulate ERK1/2 MAPK signaling. **C** WNK1 can on the one hand inhibit TGF-β signaling through physically binding to Smad2, and on the other hand activate TGF-β signaling through activating OSR1. **D** WNK1/4 positively regulate Wnt/β-Catenin signaling through interfering with the formation of the β-Catenin-GID complex and preventing the degradation of β-Catenin. In addition, the WNK1-GSK3β-SRPK1 complex induces the activation of SRPK1. In the nucleus, SRSF1 induces alternative splicing of tumor-related Rac1b, whose activation is retained by nuclear SRPK1.

In addition to regulating ion transport, WNKs are associated with other signaling pathways that play critical roles during cancer progression, including Akt, MAPK, TGF-β, GSK3β, and Wnt signaling pathways (Fig. 5).

### Akt signaling

Akt serves as an upstream activator of the WNK1-SPAK/OSR1 axis through the phosphorylation of WNK1 at residues Thr-58 and Thr-60 [71–75]. In lung cancer (LC), the Akt-WNK1-SPAK/OSR1 axis is activated by factors in the tumor microenvironment (TME) [74, 75]. Kynurenine (Kyn) is the metabolite of tryptophan and is produced by synthetizing enzyme tryptophan 2,3-dioxygenase 1 (TDO1) and indoleamine 2,3-dioxygenase 1 (IDO1) [76]. In LC-associated fibroblasts (LCAF), the activity of TDO1 and IDO1 is increased by a carbohydrate-binding protein Galectin-1, promoting LCAF-derived Kyn in the TME and further activating Akt-WNK1 signaling in cancer cells [75]. In addition to Kyn, Secreted protein acidic and rich in cysteine (SPARC), a matricellular glycoprotein that plays a vital role in the TME, can also induce Akt and WNK1 phosphorylation in LC cells [74]. The activation of the Akt-WNK1-SPAK/OSR1 axis upregulates mesenchymal markers Snail and N-cadherin and downregulates epithelial markers E-cadherin, thus promoting cancer EMT [74]. A subunit of CoA ligases Acyl-CoA medium-chain synthetase-3 (ACSM3), is found to suppress the migration and invasion of HCC cells via inhibiting the phosphorylation of WNK1 and Akt [77]. However, its expression is downregulated in HCC, and restoring its expression could inhibit WNK1/Akt-induced tumor metastasis [77].

### MAPK signaling

WNKs play a critical role in regulating MAPK signaling pathways, including ERK1/2, JNK, p38 and ERK5 MAPK signaling. In Hela cell lines, WNK1 can phosphorylate the N terminal of MAP kinase kinase kinase (MEKK)2/3 under the stimulation of EGF, leading to the activation of downstream ERK5 MAPK signaling [78, 79]. In prostate cancer (PCa), a stromal transcription factor Forkhead box F1 (FOXF1) is identified as an activator of WNK1-ERK5 signaling by binding to the −4458/−4471 region of the *Wnk1* promoter and transcriptionally activating it [80]. In vivo experiments in murine orthotopic PCa models further confirmed that the FOXF1-WNK1-ERK5 axis contributes to tumor growth and peritoneal metastasis, which can be inhibited by knockdown of *ERK5* or *WNK1* [80]. In HCC, WNK1-ERK5 signaling is activated by NKCC2, and the details are shown in part “NKCC” [61].

WNKs have a complex relationship with ERK1/2 MAPK signaling. WNK1 can promote the proliferation and migration of neural progenitor cells by activating ERK1/2 [81]. However, WNK1 is also found to inhibit ERK1/2 MAPK signaling in HEK-BKα cells, which positively regulates the activity of Ca^2+^-activated K^+^ channels [82]. For WNK4, two findings have demonstrated that WNK4 activates ERK1/2 to inhibit the activity of sodium chloride cotransporter, and this effect is independent of SPAK/OSR1 [83, 84]. However, the role of WNK1/4-ERK1/2 signaling in cancer is unclear.

WNK2 negatively regulates ERK1/2 MAPK signaling in cancer. Mechanistically, WNK2 induces the inhibitory phosphorylation of MAP kinase kinase (MEK)1 and interferes with its phosphorylation at residue Ser-298, a key site for MEK1 to activate ERK1/2 in response to EGF stimulation [85]. Depletion of *WNK2* in HeLa cells can help cells to pass the G1 phase cell cycle checkpoint and promote cell proliferation [85]. In HCC, loss-of-function mutation of *WNK2* leads to the activation of ERK1/2 MAPK signaling, contributing to tumor growth and pulmonary metastasis [18]. In cervical cancer, miR-18a is found to directly target and inhibit WNK2, leading to the activation of ERK1/2-PD-L1 signaling and promoting tumor growth and invasion [86]. In GC, WNK2 can inhibit the tumorigenicity of cancer cells by preventing the phosphorylation of ERK1/2 and p38. However, its expression is downregulated by LINC00858-mediated promoter methylation [87].

### TGF-β signaling

WNK1 is a dual regulator of TGF-β signaling in cancer. WNK1 can directly bind to and phosphorylate Smad2, a downstream protein activated by the TGF-β receptor [88]. However, WNK1-induced Smad2 phosphorylation at residue Ser-465 interferes with the TGF-β-induced phosphorylation, nuclear translocation and transcriptional activity of Smad2, thereby inhibiting TGF-β signal transduction in cancer cells [88]. On the contrary, WNK1-activated OSR1 can phosphorylate Smad2/3 at residues Thr-220/179 and enhance their activity in cancer cells, which activates pro-EMT transcription factors and further promotes the expression of TGF-β1 in an autocrine manner [89]. Both in vitro and in vivo experiments confirmed that the WNK1-OSR1-Smad2/3-TGF-β1 axis contributes to cancer progression by promoting EMT and metastasis [89].

### GSK3β and Wnt signaling

GSK3β is a serine/threonine protein kinase whose enzymatic activity is regulated via phosphorylation of its Tyr-216(active) and Ser- 9(inactive) residues [90]. In drosophila and human CRC cells, GSK3β is found as a downstream effector of WNK signaling, which is independent of the catalytic activity of WNKs [91, 92]. One mechanism is that WNK1 can physically interact with GSK3β to prevent its inhibitory phosphorylation at Ser-9, thereby promoting the assembly of a protein kinase complex consisting of WNK1, GSK3β, and serine-arginine protein kinase 1 (SRPK1) [92]. GSK3β then phosphorylates and activates SRPK1, which subsequently induces the phosphorylation of a splicing factor SRSF1 and its nuclear translocation [92]. In the nucleus of CRC cells, SRSF1 induces the alternative splicing event that generates Rac1b, a tumor-specific RNA splicing variant of the GTPase that is associated with the *BRAF*^*V600E*^ mutation of CRC [92].

The negative regulation of canonical Wnt signaling in cancer depends on the GSK3β-induced phosphorylation and the E3 ubiquitin ligase-induced ubiquitination of β-Catenin [93]. A recent study has found that WNK1 and WNK4 positively regulate the Wnt signaling in CRC cells through attenuating the interaction between β-Catenin and the glucose-induced degradation-deficient (GID) complex, a kind of E3 ubiquitin ligase [94]. The use of WNK inhibitors could induce β-Catenin degradation and inhibit Wnt signaling, thus suppressing the xenograft tumor growth of CRC [94].

### WNK1 regulates tumor angiogenesis

**Fig. 6:**
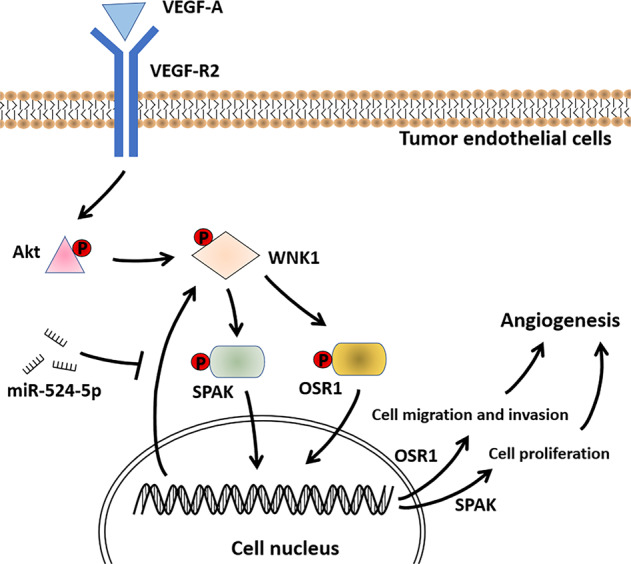
WNK1 induces tumor angiogenesis. VEGFA-VEGFR2 signaling activates Akt signaling to induce WNK1 activation in tumor endothelial cells. WNK1-OSR1 signaling promotes cell migration and invasion, while WNK1-SPAK signaling promotes cell proliferation, to promote tumor angiogenesis. MiR-524-5p can inhibit WNK1 expression to prevent tumor angiogenesis.

WNK1 rather than other WNK family members is required for angiogenesis (Fig. 6). The expression of *WNK2* and *WNK4* are undetectable in human endothelial cells (hECs) [95]. A low expression level of *WNK3* is detected in hECs, while *WNK3* knockout mice did not have any angiogenesis defects [95, 96]. In contrast, the activation of WNK1-OSR1 signaling is critical for angiogenesis and cardiac formation, and endothelial-specific depletion of either *WNK1* or *OSR1* in mice results in defective angiogenesis and cardiovascular development [97, 98]. Knockdown of *WNK1* in hECs leads to the reduced expression of *VEGF-A* and pro-EMT genes such as *MMP2*, *MMP9*, *Slug*, and *ZEB1* [95]. Interestingly, depletion of *OSR1* inhibits the migration of hECs, while depletion of *SPAK* inhibits the proliferation of hECs. This suggests that WNK1-OSR1 signaling and WNK-SPAK signaling may affect angiogenesis through different patterns [95].

The rescue experiments in zebrafish have revealed that VEGF/VEGF-R2 signaling activates WNK1 through PI3K-Akt signaling, thus promoting angiogenesis [99]. In zebrafish models of CRC and HCC, treatment with WNK1-SPAK/OSR1 axis inhibitors WNK463 and Closantel significantly reduced the expression of *CCND1* and *MMP9*, and inhibited tumor metastasis and angiogenesis [100]. Compared with VEGFR inhibitor PTK787, WNK463 and Closantel had shown stronger anti-tumor effects, indicating their value for inhibiting tumor angiogenesis [100].

Recently, miR-524-5p is found to inhibit angiogenesis in CRC by targeting WNK1 [101]. Incubated with tumor cell-conditioned medium from WNK1-knockdown and/or miR-524-5p-overexpressed CRC cells, the migratory ability and the number of capillary-like structures in hECs were significantly reduced. Moreover, overexpression of miR-524-5p could also markedly inhibit tumor growth and microangiogenesis in a murine CRC model [101].

### WNK2 inactivation contributes to cancer progression

The low expression levels of *WNK2* have been found in several kinds of cancer, mainly induced by aberrant methylation in the 3’CpG island of gene promoter [13, 15, 87, 102–104]. In brain tumors, *WNK2* methylation specifically occurs in meningiomas (5 of 6 grade II and 5 of 7 grade III cases in a study) and adult gliomas (29 of 166 cases in a study) [15, 102]. Epigenetic inhibition of *WNK2* correlates significantly with co-deletion of chromosome arms 1p/19q, a molecular genetic signature of oligodendroglial tumors [13]. In a study of 736 HCC patients, somatic mutation and copy number loss occurred in 39 and 200 cases, respectively, and the low expression of WNK2 is associated with poor OS and early tumor recurrence [18]. In addition, hypermethylation of the *WNK2* gene also contributes to the development of HBV-related HCC [103].

A transcriptional repressor chromobox protein homolog 8 (CBX8) can directly bind to the *WNK2* promotor and repress its activity in human cancers [105]. In addition, several kinds of ncRNAs are involved in the inactivation of WNK2 to affect cancer progression (see details in part “WNKs and ncRNAs in cancer”) [87, 106–109]. Loss of WNK2 expression leads to the activation of oncogenic signaling pathways, such as ERK1/2, JNK-MMP2/9 and Rac1, thus enhancing tumor growth and metastasis [15, 18, 85, 86, 105, 110].

### WNKs regulate autophagy in cancer

Autophagy is a type II programmed cell death and has roles in both promoting and inhibiting tumor growth [111]. WNK1 is found to repress autophagy in Hela and A549 cancer cell lines through complex mechanisms: Through inhibiting AMPK signaling, WNK1 inhibits the phosphorylation and activity of the autophagy kinase ULK1, a key protein complex for the initiation step of autophagy [112]. In addition, WNK1 can directly bind to UVRAG, a component of the PI3KC3 complex that is downstream of ULK1. The interaction of WNK1 and UVRAG blocks the PI3KC3 complex, leading to autophagy inhibition [112]. Notably, knockdown of *SPAK* rather than *OSR1* stimulates autophagy, indicating WNK1 may also inhibit autophagy through SPAK [112].

**Fig. 7:**
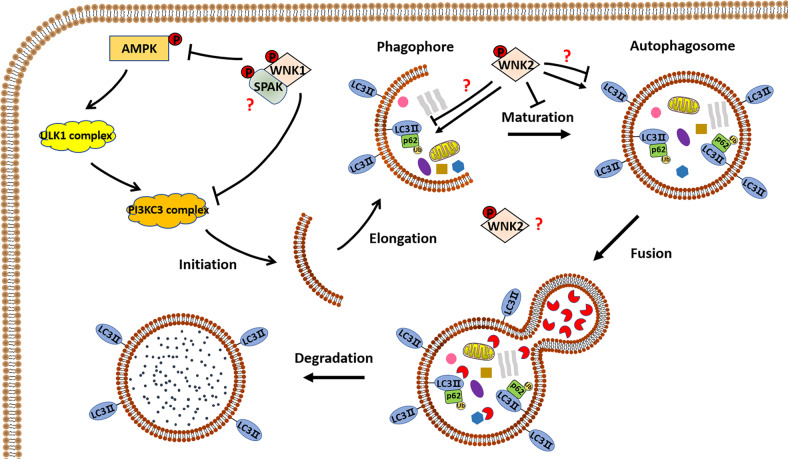
WNK1 and WNK2 regulate autophagy in cancer cells. WNK1 inhibits the initiation of autophagy through inhibiting the activation of MAPK-ULK1 signaling and interacting with UVRAG (not shown in the figure) of the PI3KC3 complex. SPAK is also involved in the negative regulation of autophagy, in an unclear manner. WNK2 may control the progress of autophagy through regulating the expression of p62 and LC3B. Both promoting and inhibitory effects of WNK2 on p62 and LC3B have been found, but the exact mechanisms remain unknown.

The role of WNK2 is controversial in regulating autophagy in cancer. In BC cell lines MCF-7, knockdown of *WNK2* promotes the accumulation of the autophagic substrate p62 and leads to defective maturation of autophagosomes, thus inhibiting autophagy [113]. In CRC cell lines HT-29, CT26, SW480, and HCT116, inhibition of WNK2 significantly reduced the expression of autophagic markers Beclin-1 and LC3BII/I, suggesting that WNK2 is a positive regulator of autophagy [106]. However, in glioma cell lines A172 and human leukemia cell lines K562, WNK2 is found to markedly downregulate the expression of LC3B and p62 and inhibit autophagy [114, 115]. These findings indicate the mechanisms by which WNK2 regulates autophagy may differ in different types of cancer; however, the exact mechanisms have not been well elucidated (Fig. 7).

### WNKs and ncRNAs in cancer

NcRNAs are a class of RNA molecules that do not encode proteins and function to regulate gene expression [116]. So far, several kinds of ncRNAs have been identified to regulate WNKs in cancer (Table 1). MicroRNAs (miRNAs) are small ncRNAs that post-transcriptionally repress gene expression via binding to the 3′-untranslated region of target mRNAs [116]. MiR-93, miR-130a, miR-210a, miR-524a, and miR-620 are found to directly target oncogenic WNK1/3 or antitumor WNK2, thus affecting cancer progression [101, 107, 109, 117–119]. However, the inhibitory effect of these miRNAs on WNK expression can be released by the “sponge effect” of long non-coding RNAs (lncRNAs) and circular RNAs (circRNAs), such as lncRNA FTX, lncRNA H19 and circRNA circ_0001666 [109, 118, 119]. In addition, recent studies have found that lncRNA LINC00858 can directly repress the expression of WNK2 in cancer cells and contribute to cancer progression [87, 106, 108]. Mechanistically, LINC00858 binds to the *WNK2* promotor, on the one hand enhancing promoter methylation, and on the other hand recruiting and activating the transcription factor HNF4α, a negative regulator of WNK2 [87, 106, 108]. Future studies should further explore the potential mechanisms of ncRNAs regulating WNKs and the roles of ncRNAs in WNK-involved biological processes during cancer progression.

**Table 1:** Non-coding RNAs that regulate the expression of WNKs in cancer.

Non-coding RNAs	Function	Cancer type	Function	Reference
miR-93	Tumor suppressive	Triple-negative breast cancer	miR-93 inhibits cancer cell invasion in vitro by targeting WNK1	[117]
lncRNA FTX miR-320a	Oncogenic Tumor suppressive	Retinoblastoma	FTX promotes the proliferation, invasion and migration of cancer cells in vitro and promotes tumor growth in vivo by targeting the miR-320a/WNK1 axis	[118]
miR-370	Oncogenic	Breast cancer	MiR-370 promotes cancer cell proliferation and promotes tumor growth in vivo by targeting WNK2	[107]
miR-524	Tumor suppressive	Colorectal cancer	miR-524 inhibits cancer cell proliferation and angiogenesis in vitro and inhibits tumor growth in vivo by targeting WNK1	[101]
LINC00858	Oncogenic	Colorectal cancer	LINC00858 inhibits the apoptosis, senescence, and autophagy of cancer cells in vitro by targeting WNK2	[106]
			LINC00858 promotes the proliferation, invasion and migration of cancer cells and angiogenesis in vitro and promotes tumor growth and angiogenesis in vivo by targeting WNK2	[108]
		Gastric cancer	LINC00858 promotes the growth, migration and invasion of cancer cells in vitro and inhibits tumor growth in vivo by activating WNK2/MAPK signaling pathway	[87]
Circ_0001666 miR-620	Tumor suppressive Oncogenic	Breast cancer	Circ_0001666 inhibits the proliferation, migration and invasion of cancer cells in vitro and inhibits tumor growth in vivo by suppressing miR-620/WNK2 axis	[109]
lncRNA H19miR-130a	Oncogenic Tumor suppressive	Non-small cell lung cancer	H19 induces the radiotherapy resistance of cancer cells in vitro by targeting the miR-130a/WNK3 axis	[119]

### Potential pharmacological treatments for blocking WNK-OSR1/SPAK signaling in cancer

To date, several compounds/drugs have been designed for the treatment of diseases caused by the activation of WNK-OSR1/SPAK signaling such as hypertension, and they are WNK inhibitors, OSR1/SPAK inhibitors and WNK-SPAK/OSR1 binding disruptors (Table 2) [66, 89, 92, 94, 100, 120–130].

**Table 2:** Known drugs that block WNK-SPAK/OSR1 signaling.

Drug	Structure	Targeted strategy	Experimental model	Function	Reference
WNK463		WNK inhibitor	BC CRC CRC and HCC	Inhibits cancer cell proliferation in vitro. Inhibits cancer cell invasion in vitro and attenuates tumor growth and metastatic burden in a NSG mice model of BC. Inhibits WNK1 expression in CRC cells. Inhibits tumor growth, metastasis and angiogenesis in zebrafish xenograft models	[89, 120, 92, 100]
			Hypertension	Decreases blood pressure and increases electrolyte excretion in spontaneously hypertensive rats	[121]
			Pulmonary arterial hypertension	Improve right ventricular function and survival in rats by combating metabolic dysregulation	[122]
WNK-IN-11		WNK inhibitor	Primary bone marrow-derived cells	Inhibits WNK1-induced NLRP3 inflammasome activation and pyroptosis in macrophages	[123]
			Mouse cortical collecting duct cells	Inhibits WNK1-induced translocation of TRPV4 channel to the plasma membrane	[124]
Closantel		SPAK/OSR1 inhibitor	Bone cancer HCC Lymphoma, cervical cancer, PCa, HCC and CRC	Attenuates bone cancer pain in rats. Inhibits cancer cell viability in vitro. Inhibits tumor growth, metastasis and angiogenesis in zebrafish xenograft models	[66, 125, 100, 126]
Rafoxanide		SPAK/OSR1 inhibitor	HCC BC	Inhibits cancer cell viability in vitro. Inhibits cancer cell proliferation and EMT in vitro	[125, 89]
Verteporfin		SPAK/OSR1 inhibitor	No reports	–	–
STOCK1S-14279		SPAK/OSR1 inhibitor	No reports	–	–
ZT-1a		WNK-SPAK/OSR1 binding disruptor	Ischemic stroke	Inhibits cerebral edema and infarct and improves neurological function after ischemic stroke in Wistar rats. Increases ChP blood–CSF barrier integrity and reduces the neuroinflammation responses after ischemic stroke in C57BL/6J mice. Reduces brain lesion size and improves neurological function in C57BL/6J mice	[130, 127, 128]
STOCK2S-26016		WNK-SPAK/OSR1 binding disruptor	BC	Inhibits the proliferation, migration, invasion and chemotherapy resistance of cancer cells in vitro	[129]
STOCK1S-50699		WNK-SPAK/OSR1 binding disruptor	CRC	Inhibits cancer cell proliferation in vitro and inhibits xenograft tumor growth in a Balb/c mice model of CRC	[94]

*BC* breast cancer, *CRC* colorectal cancer, *HCC* hepatocellular carcinoma, *NLRP3* NOD-like receptor thermal protein domain associated protein 3, *TRPV4* transient receptor potential cation channel subfamily V member 4, *PCa* prostate cancer, *EMT* epithelial–mesenchymal transition, *ChP* choroid plexus, *CSF* cerebrospinal fluid.

### WNK inhibitors

WNK463 is an orthosteric ATP-competitive WNK inhibitor that interacts with the hinge region of the ATP binding site of WNKs [121]. It is the first orally pan-WNK inhibitor and exhibits both high kinase selectivity and low nanomolar affinity [121]. Oral dosing of WNK463 in rats led to decreased phosphorylation of OSR1 and SPAK and reduced blood pressure [121]. In BC, CRC and HCC, WNK463 treatment exhibits potent anti-tumor effects both in vivo and in vitro, inhibiting tumor growth, metastasis and angiogenesis [89, 92, 100, 120]. However, the development of WNK463 is discontinued due to preclinical safety issues in rats at 1–10 mg/kg doses, including elicited ataxia and breathing difficulties [121, 130].

Compared with WNK463, WNK-IN-11 is an allosteric non-ATP-competitive WNK inhibitor, showing a reasonable pharmacokinetic profile with moderate clearance in rats [131]. It has been applicated to inhibit WNK1 in primary bone marrow-derived cells and muse cortical collecting duct cells [123, 124]; however, its effects have not been evaluated in tumors.

### SPAK/OSR1 inhibitors

Closantel, Rafoxanide, Verteporfin and STOCK1S-14279 are allosteric non-ATP-competitive SPAK/OSR1 inhibitors. Mechanistically, Closantel, Rafoxanide and STOCK1S-14279 can bind to a secondary pocket in the CCT domain of SPAK/OSR1 and inhibit SPAK/OSR1 activity [132]. Instead, Verteporfin can bind to an allosteric site adjacent to the kinase domain of SPAK/OSR1 and exhibit potent inhibition of OSR1 T185E and SPAK T233E [133].

Closantel and Rafoxanide have been applicated to block WNK-OSR1/SPAK signaling in cancer. Treatment of Closantel could attenuate bone cancer pain in rats, and it could also inhibit tumor growth and angiogenesis in zebrafish models of several types of cancer [66, 100, 126]. Treatment of Rafoxanide, instead, could inhibit cancer cell survival and inhibit the expression of pro-EMT transcription factors *Twist1*, *Snail* and *Slug* in cancer cells [89, 125].

### WNK-SPAK/OSR1 binding disruptors

STOCK2S-26016, STOCK1S-50699 and ZT-1a are small-molecule inhibitors that interfere with the interaction between WNK and SPAK/OSR1 by interacting with the CCT domain of SPAK/OSR1 [130, 134]. A recent study has reported that STOCK2S-26016 treatment could inhibit the tumorigenicity of BC cells, especially inhibiting their invasive ability and enhancing their sensitivity to paclitaxel treatment [129]. In CRC, Sato et al. have found that STOCK1S-50699 treatment could inhibit the interaction between WNK1/4 and the GID complex, leading to the reduced degradation of β-Catenin and enhanced oncogenic Wnt/β-Catenin signaling [94].

ZT-1a is a novel-designed allosteric non-ATP-competitive WNK-SPAK/OSR1 binding disruptor. It has been wildly used to attenuate neuroinflammation and brain damage in pre-clinical mouse models of ischemic stroke by inhibiting WNK-SPAK/OSR1-NKCC1 axis [127, 128, 130]. Further studies should be performed to evaluate its effects in the field of cancer.

## Conclusions and perspectives

WNKs play a key role in maintaining ion homeostasis, and their dysregulation can lead to diseases such as hypertension [135]. In recent years, more and more studies have found that WNKs are overexpressed or downregulated in cancer tissues, contributing to tumor growth, metastasis and angiogenesis. Mechanistically, WNKs may regulate/be regulated by ion channels (ENaC, TRPC6 and ClC3), CCCs (NKCC1/2 and KCC2), NBCs, signaling pathways (Akt, MAPK, TGF-β, GSK3β and Wnt signaling), autophagy and ncRNAs in cancer.

The catalytic function of WNKs is mainly dependent on the phosphorylation of residues Ser-382 within the kinase domain and the subsequent phosphorylation and activation of SPAK/OSR1 [2]. In addition, WNK1 is also found to act as a scaffolding protein to recruit SPAK/OSR1 on its target proteins, such as NBCs, and this effect is dependent on the phosphorylation of residue Ser-60 [70]. Interestingly, phosphorylation of WNK1 at residue Ser-60 in cancer cells is also found to support the catalytic function of WNK1, such as inducing phosphorylation of SPAK/OSR1 and CLC3 [50, 74, 75]. Since the phosphorylation of WNKs is the key to their subsequent function, it is necessary to clarify the relationship between different phosphorylation sites and the catalytic/scaffolding function of WNKs in cancer cells.

WNK1 is the most well-studied member of the WNK family in cancer. Recently, some novel functions of WNK1 have been revealed in non-tumor cells: In macrophages, WNK1 is found to suppress the activation of the NLRP3 inflammasome, a multimeric cytosolic protein complex that induces pyroptosis and plays dual roles during cancer progression [123]. In addition, WNK1 is found to increase glucose uptake of HEK293 cells by increasing the surface expression of GLUT1, a glucose transporter that is critical for tumor metabolism [136]. It remains to be confirmed whether WNK1 and the other three WNK members participate in these mechanisms in cancer.

Blocking WNK-OSR1/SPAK signaling is an important strategy for the treatment of hypertension, and drugs have been tested in preclinical animal models. One problem with the use of these drugs in cancer is their potential side effects at high doses [121, 130]. For example, treatment of pan-WNK inhibitors may result in off-target effects, which is due to lack of selectivity for the WNK members. WNK1, 3 and 4 mainly play oncogenic roles during cancer progression. However, several studies have confirmed that WNK2 has tumor-suppressive functions in cancer, while its expression is downregulated in cancer tissues (See part “WNK2 inactivation contributes to cancer progression”). Thus, inhibition of WNK1, 3 and 4 while selectively activating WNK2 activity may be a better therapeutic strategy for cancer treatment.

Although pan-WNK inhibitors such as WNK463 have been wildly used, few compounds for targeting WNKs have been reported, with even multi-kinase inhibitors exhibiting no anti-WNK activity [11]. Among all WNK family members, highly selective WNK1 inhibitors are under development, while WNK2 and WNK3 are dark kinases whose druggability is poorly investigated [11, 137, 138]. Actually, selective inhibition of WNK family members may be feasible due to the differences in structure and molecular dynamics among different family members [137–139]. Recent studies have found that targeting the less conserved allosteric binding sites rather than the highly conserved ATP-binding sites of WNKs may achieve selectivity among WNK family members, such as an allosteric inhibitor WNK476 (WNK-IN-12) imparts high specificity against WNK1 [131, 138]. Future studies should develop more allosteric WNK inhibitors and test their efficacy and safety in preclinical animal models.

In a recent study, Jonniya et al. developed a compound meciadanol that shows high specificity and binding affinity toward WNK1 through high-throughput screening of phytochemical compounds and kinase inhibitors [137]. Specifically, they have performed ADMET analysis, molecular docking analysis, molecular dynamics simulations, and MM-PBSA analysis to identify candidate anti-WNK1 drugs [137]. These analysis methods provide a reference for screening and developing other compounds/drugs for targeting WNK family members.

In conclusion, WNKs have multiple functions in cancer, and their activities are involved in the regulation of cancer development through a series of complex mechanisms. Further studies should explore the exact functions of WNKs in cancer and develop safer and more effective drugs for targeting WNK-SPAK/OSR1 signaling.

## Data Availability

All data generated or analyzed during this study are included in this published article.
